# A synthetic peptide exerts nontolerance-forming antihyperalgesic and antidepressant effects in mice

**DOI:** 10.1016/j.neurot.2024.e00377

**Published:** 2024-05-22

**Authors:** Yongjiang Wu, Xiaofei Song, YanZhe Ji, Gang Chen, Long Zhao

**Affiliations:** a Center for Basic Medical Research, Medical School of Nantong University, Co-innovation Center of Neuroregeneration, Nantong, Jiangsu Province, China; b Key Laboratory of Neuroregeneration of Jiangsu and the Ministry of Education, Co-innovation Center of Neuroregeneration, Nantong University, Nantong, Jiangsu Province, China; c Department of Anesthesiology, Affiliated Hospital of Nantong University, Nantong, Jiangsu Province, China

**Keywords:** Chronic pain, Analgesic, Opioid receptor, Antidepressant effect, Comorbidity

## Abstract

Chronic pain is a prevalent and persistent ailment that affects individuals worldwide. Conventional medications employed in the treatment of chronic pain typically demonstrate limited analgesic effectiveness and frequently give rise to debilitating side effects, such as tolerance and addiction, thereby diminishing patient compliance with medication. Consequently, there is an urgent need for the development of efficacious novel analgesics and innovative methodologies to address chronic pain. Recently, a growing body of evidence has suggested that multireceptor ligands targeting opioid receptors (ORs) are favorable for improving analgesic efficacy, decreasing the risk of adverse effects, and occasionally yielding additional advantages. In this study, the intrathecal injection of a recently developed peptide (VYWEMEDKN) at nanomolar concentrations decreased pain sensitivity in naïve mice and effectively reduced pain-related behaviors in nociceptive pain model mice with minimal opioid-related side effects. Importantly, the compound exerted significant rapid-acting antidepressant effects in both the forced swim test and tail suspension test. It is possible that the rapid antihyperalgesic and antidepressant effects of the peptide are mediated through the OR pathway. Overall, this peptide could both effectively provide pain relief and alleviate depression with fewer side effects, suggesting that it is a potential agent for chronic pain and depression comorbidities from the perspective of pharmaceutical development.

## Introduction

Chronic pain is a major cause of human suffering and disability worldwide [1]. Typically, pathogenic infections, trauma, metabolic dysfunction, or tumor invasion provoke inflammatory tissue damage or nerve injury, thus causing chronic pain [2,3]. However, the pathogenesis of chronic pain is not well understood, and thus far, development of effective therapeutics for chronic pain relief remains an unmet clinical need [4]. Additionally, it is important to note that patients who suffer from chronic pain are at greater risk of experiencing anxiety, depression, and sleep dysfunction, which further exacerbates pain severity [5,6]. Thus, timely, effective treatment for chronic pain and comorbidities is essential and needs far more attention [7].

Opioidergic drugs (e.g., morphine and fentanyl) are the most effective prescribed analgesics for alleviating moderate to severe pain conditions, and they act mainly through *μ*-opioid receptors (*μ*ORs) [8,9]. However, extended opioid administration for treating and eradicating chronic pain (neuropathic pain) is largely unsatisfactory due to their relatively low efficacy and severe adverse effects (e.g., tolerance, respiratory depression, and addiction) [10]. This situation reduces medication adherence, augments the risk of opioid misuse, and further feeds the ‘‘opioid crisis’’ [10,11]. Safer and more effective opioid analgesics with novel chemical structures are urgently needed to combat chronic pain and mitigate the “opioid epidemic”.

Analgesic effects can also be mediated by activation of the *δ*-opioid receptor (*δ*OR) [12] and *κ*-opioid receptor (*κ*OR) [13]. However, thus far, no selective *δ*OR or *κ*OR agents are approved for marketing due to limited analgesic potency and their own distinct profile of side effects (e.g., convulsive effects for *δ*OR agonists; dysphoria and sedation for *κ*OR agonists) [14]. In recent years, many studies have focused on the design and clinical development of novel analgesics that can simultaneously target two or more opioid receptors, thus achieving additional analgesia and at the same time having a more favorable side effect profile [14,15]. The approach described here offers possibilities to further overcome the existing intrinsic limitations of prescription opioid analgesics. In addition, intensive efforts in peptide drug discovery studies confirmed that following central or peripheral administration, peptide-based small molecules exhibited potent and durable analgesia with reduced opioid-related side effects in multiple preclinical pain models, including inflammatory pain (e.g., induced by Complete Freund's adjuvant, CFA) and neuropathic pain (induced by nerve injury, diabetes, or chemotherapy) [[16], [17], [18]].

In the present study, we developed a new peptide-based ligand that exhibits an opioid antihyperalgesic and antidepressant effect (reversible by opioid antagonist) with fewer deleterious effects. Studies in various mouse models indicated that this new molecular entity has considerable therapeutic potential in the treatment of chronic pain and depression. In addition, knowledge of bi/multifunctional ligands will be beneficial for the rational design of next-generation analgesics.

## Materials and methods

### Reagents

Peptide (purity ≥98%) in this study was designed by our laboratory and were synthesized from GL Biochem Ltd. (Shanghai, China). Naloxone hydrochloride (Ab120074), nor-Binaltorphimine (nor-BNI, Ab120078) and CTOP (ab120417) were purchased from Abcam. Naltrindole hydrochloride (NTI, HY-101177) and capsaicin (HY-10448) was obtained from MCE (Shanghai, China). *λ*-carrageenan (22049) and CFA (F5881) were purchased from Sigma-Aldrich (St. Louis, MO).

### Animals and drug administration

All behavioral experiments were conducted using male ICR mice (8 weeks, 6–8 mice/group) and were approved by the Animal Care and Use Committee of Nantong University. The figure legends provided a comprehensive account of the number of mice utilized in the study. All mice were purchased from the Experimental Animal Center of Nantong University (Jiangsu, China). Mice were housed under a 12 ​h light/dark cycle. Food and water were available ad libitum. All drugs were dissolved in physiological saline. The intrathecal (i.t.) injection was performed in conscious mice as previously described [16]. Carrageenan/CFA was injected into the left hind paw of mice via the intraplantar (i.pl.) administration to induce inflammatory pain. The injection volumes were 10 ​μl (for i.t.) and 20 ​μl (for i.pl.), respectively.

### Analgesic activity evaluation

#### Von Frey test for mechanical pain

Mechanical nociceptive thresholds in mice was evaluated using the up-down method with a set of von Frey filaments (0.16, 0.4, 0.6, 1.0, 2.0 ​g). The experimental procedure was carried out in a blinded manner as previously described [19]. The mice were allowed to acclimate to the experimental apparatus for two consecutive days before the experiments were initiated. After that, basal mechanical pain threshold in naïve mice were assessed prior to modeling or pharmaceutical intervention. On the test day, the central plantar surface of left mouse hindpaw was stimulated perpendicularly by von Frey hairs through the elevated metal mesh floor, and the withdrawal response of left hindpaw was recorded.

#### Capsaicin-induced spontaneous pain

The procedure of the capsaicin-induced paw licking test was performed in a blinded manner following our previously established protocol [16]. Mice were kept in transparent acrylic boxes on an elevated metal mesh floor and allowed to acclimate to the environment for 30 ​min. Afterward, the drug was previously administered by i.t. injection. After 5 ​min, capsaicin (1.6 μg/paw, 10 ​μl) was injected into the left mouse hindpaw by intraplantar injection. Subsequently, the mouse was put back into the box, and the duration of nocifensive behavior (licking and biting the injected paw) was recorded for 5 ​min.

#### Carrageenan-induced acute inflammatory pain

For the carrageenan-induced acute inflammation model, carrageenan (2%, 20 ​μl, i.pl.) was injected intradermally into the hind pad of each mouse one day in advance. The paw withdrawal threshold of the left hindpaw was measured using the von Frey test before and after the interventions at different time points.

#### CFA-induced persistent inflammatory pain

A single dose of CFA (20 ​μl, i.pl.) injection induced persistent inflammatory, thus provoking edema and mechanical allodynia of the injected paw [16]. Four days after CFA injection, mechanical allodynia was assessed before and after the interventions.

#### Chronic sciatic nerve constriction injury (CCI)-induced peripheral neuropathic pain

The CCI model was generated to evaluate the analgesic effects of the compounds on neuropathic pain as previously described [16]. Briefly, CCI surgical procedures were conducted under isoflurane anesthesia. An incision was made to expose the sciatic nerve, and 3 pieces of 8/0 silk were loosely tied around the sciatic nerve. On postsurgical Day 14, the mice were subjected to behavioral testing for neuropathic pain.

### Evaluation of antidepressant-like effects

#### Forced swim test (FST)

The FST was performed in a plexiglass cylinder (height, 30 ​cm; inner diameter, 16 ​cm) as previously described [16]. The mice were gently placed in water, forced to swim, and recorded for a period of 6 ​min using a video camera. The immobility duration was recorded during the last 4 ​min of the 6 ​min testing period in a double-blinded manner.

#### Tail suspension test (TST)

The TST was performed as described by Zhang et al. [6]. In brief, the mice were suspended approximately 50 ​cm from the ground by securing their tails at a point 1 ​cm from the tip. The behavioral responses of the mice were video recorded for a period of 6 ​min. The immobility duration during the last 5 ​min was measured in a double-blinded manner.

### Adverse effects assessment

#### Development of tolerance

To assess the potential analgesic tolerance of the test peptide, the CFA-induced chronic inflammatory pain model was used as described previously [16]. CFA-model mice were i. t. administered the drug (100 ​nmol) once daily (9 a.m.) for 7 consecutive days. Thereafter, the mechanical threshold responses were measured at 0.5, 1, 1.5, 2, and 3 ​h after dosing during the first and seventh days.

#### Fecal sample analysis

Fecal sample analysis of mice was performed to evaluate the effects of drug treatment on gastrointestinal function, as we previously reported [16,20]. Naïve mice were fasted for 16 ​h and were provided with ad libitum access to water. Afterward, the fecal pellets of each mouse were collected and recorded for 1 ​h after drug administration.

#### Body temperature measurement

The body temperature of the mice (rectal temperature) was monitored during the awake state using a rectal electrothermometer (FT3400) according to a previous study [21]. The ambient temperature was controlled at 21 ​°C. A rectal temperature probe was inserted 2.5 ​cm deep into the rectum. Body temperature was measured at 0.5 and 1 ​h after peptide administration.

### Analysis

The data in this work were analyzed by GraphPad Prism 8.0 software and are expressed as the means ​± ​SEM. For statistical comparisons, Student's t-test or one-way or two-way (repeated-measures, RM) ANOVA followed by Bonferroni post hoc correction was used. P ​< ​0.05 indicated statistical significance.

## Results

### The peptide increases the pain threshold and attenuates nocifensive behavior

**Fig. 1:**
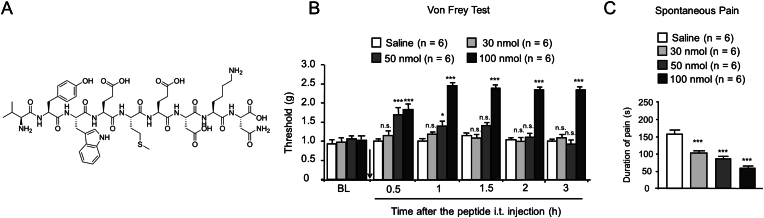
The peptide increases pain threshold in naïve mice and attenuates capsaicin-induced pain-like behaviors. (A) The chemical structure of the test peptide. (B) Von Frey test is used for assessing the basal mechanical pain of naïve mice after i. t. injection of drug. Mean ​± ​SEM, n ​= ​6. ∗p ​< ​0.05 or ∗∗∗p ​< ​0.001 versus Saline group, n. s., no significance. Two-way RM ANOVA followed by the Bonferroni's test. (C) The test peptide inhibits capsaicin-induced spontaneous pain. Mean ​± ​SEM, n ​= ​6. ∗∗∗p ​< ​0.001 versus Saline group. One-way ANOVA followed by the Bonferroni's test.

Fig. 1A shows the chemical structure of the synthetic peptide. The molecular weight is 1213.3 ​g/mol, and the amino acid sequence is Val^1^-Tyr^2^-Trp^3^-Glu^4^-Met^5^-Glu^6^-Asp^7^-Lys^8^ -Asn^9^-OH. First, we examined whether this synthetic peptide modulates pain perception at the spinal level in naïve mice. Our behavioral assessment revealed that mice displayed relatively longer and pronounced increases in paw withdrawal thresholds after i.t. administration of the test peptide (1 ​h for 50 ​nmol; more than 3 ​h for 100 ​nmol; Fig. 1B). At the lowest dose (30 ​nmol), no significant changes were detected in the mechanical withdrawal thresholds.

Capsaicin-induced acute pain was subsequently evaluated by determining the duration of spontaneous pain behavior (flinching/licking) 5 ​min after capsaicin dosing. As shown in Fig. 1C, the i.t. administration of the test peptide prior to i.pl. capsaicin injection attenuated the nociceptive response in mice in a dose-dependent manner (F_3,20_ ​= ​26.82, p ​< ​0.0001). The peptide was effective at alleviating capsaicin-induced pain behavior, with an ED_50_ value of 55 ​nmol.

### The test peptide relieves acute and chronic pain

**Fig. 2:**
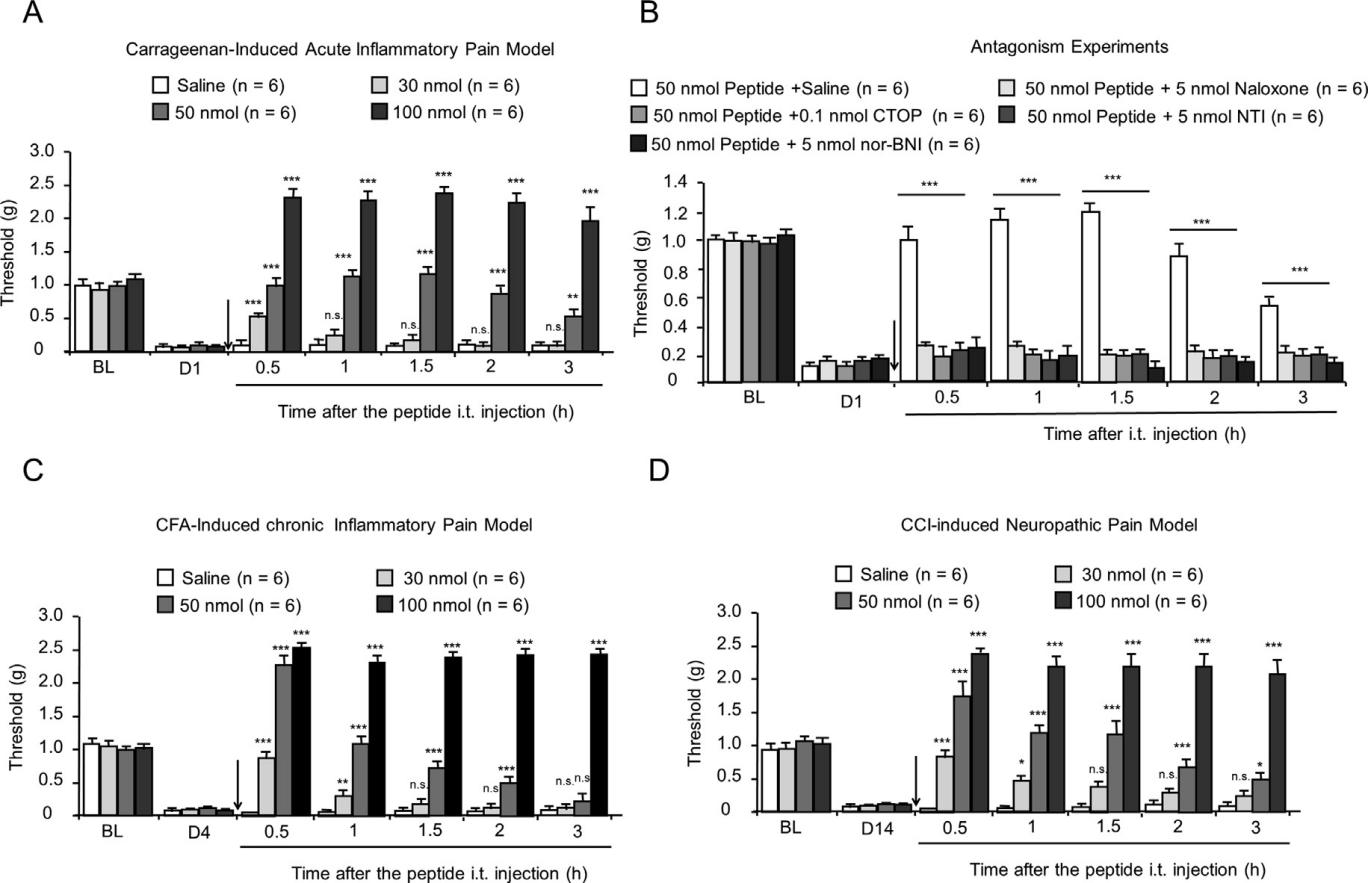
The test peptide relieves irritant substances-induced inflammatory pain and CCI-induced neuropathic pain. (A) Carrageenan-induced acute inflammatory pain. Mean ​± ​SEM, n ​= ​6. ∗∗p ​< ​0.01, ∗∗∗p ​< ​0.001 versus Saline group, n. s., no significance. Two-way RM ANOVA followed by the Bonferroni's test. (B) Effects of OR antagonists on the antihyperalgesic of the test peptide. Mean ​± ​SEM, n ​= ​6. ∗∗∗p ​< ​0.001 versus Peptide group, n. s., no significance. Two-way RM ANOVA followed by the Bonferroni's test. (C) CFA-induced chronic inflammatory pain. Mean ​± ​SEM, n ​= ​6. ∗p ​< ​0.05, ∗∗p ​< ​0.01, ∗∗∗p ​< ​0.001 versus Saline group, n. s., no significance. Two-way RM ANOVA followed by the Bonferroni's test. (D) CCI-induced persistent neuropathic pain. Mean ​± ​SEM, n ​= ​6. ∗p ​< ​0.05, ∗∗p ​< ​0.01, ∗∗∗p ​< ​0.001 versus Saline group, n. s., no significance. Two-way RM ANOVA followed by the Bonferroni's test.

Next, the effect of the synthetic peptide on nociceptive models involving chemical sensitization was detected using a carrageenan-induced acute inflammatory pain model. Plantar injection of *λ*-carrageenan evokes acute peripheral inflammation, resulting in marked edema and mechanical allodynia in the Von Frey withdrawal responses of the injected paw. In the present study, the intrathecal injection of the test peptide exerted a clear dose-dependent antihyperalgesic effect (Fig. 2A, F_3,31_ ​= ​203.9, p ​< ​0.0001).

To investigate the possible mechanism of action of the test peptide in modulating nociceptive behaviors, the nonselective OR antagonist naloxone, selective *μ*OR antagonist CTOP, selective *δ*OR antagonist NTI, and selective *κ*OR antagonist nor-BNI were used in a carrageenan-induced inflammatory pain model. As shown in Fig. 2B, the antihyperalgesic effect in the inflamed paw induced by the test peptide was significantly reversed by coinjection of naloxone (5 ​nmol), CTOP (0.1 ​nmol), NTI (5 ​nmol), or nor-BNI (5 ​nmol).

Previous research has confirmed that the efficacy of opioid analgesics in managing chronic pain is considerably lower than their effectiveness in addressing acute pain [22]. As shown in Fig. 2C and D, the test peptide showed significant and long-lasting antihyperalgesic effects in both the CFA-induced chronic inflammatory pain model and the CCI-induced neuropathic pain model.

### The test peptide exerts an antidepressant-like effect

**Fig. 3:**
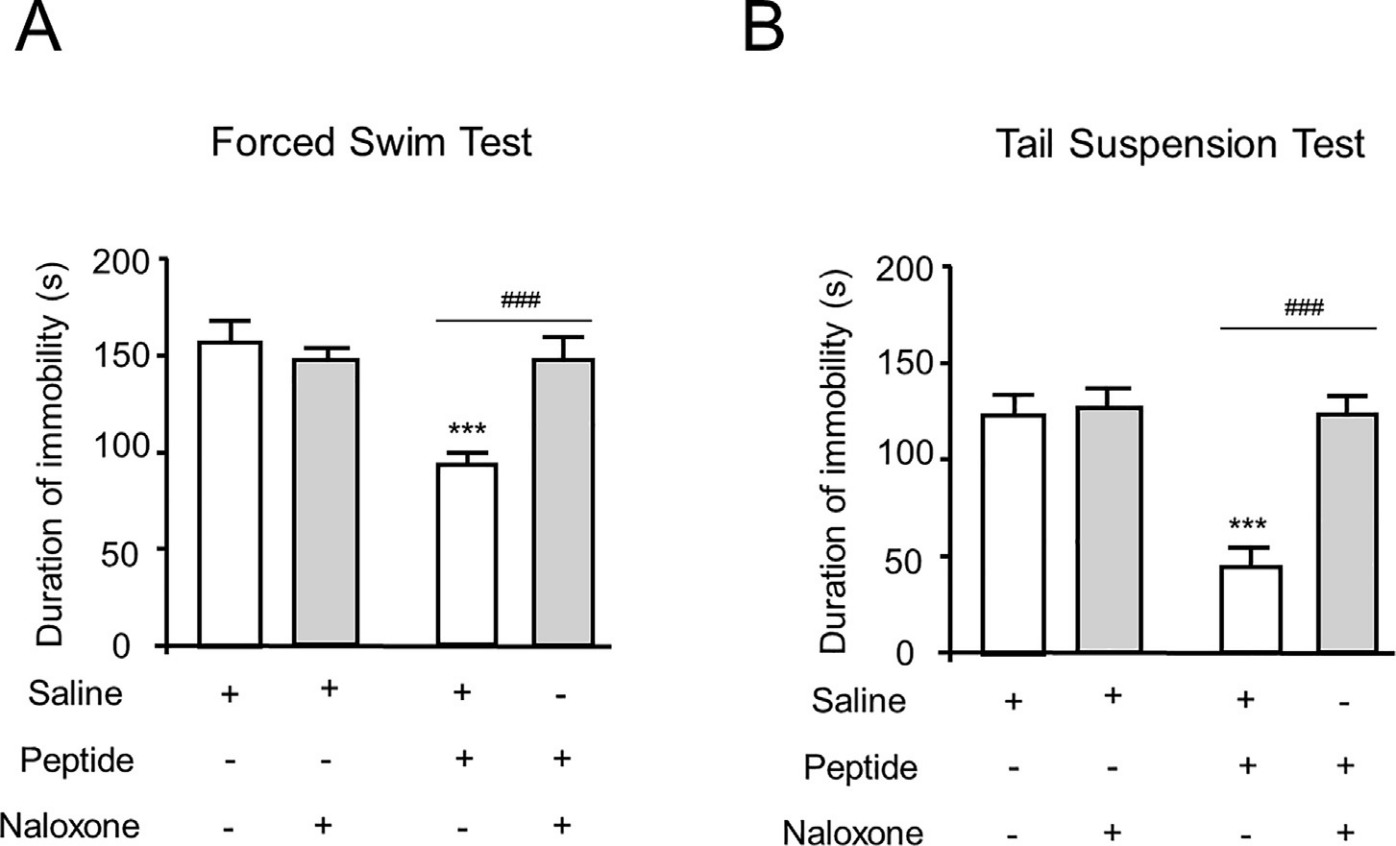
The test peptide exerts an antidepressant effect after i. t. injection in (A) forced swim test. (B) tail suspension test. The injected dose is 100 nmol/mouse for peptide, and 5 nmol/mouse for naloxone. Mean ​± ​SEM, n ​= ​8. ∗∗∗p ​< ​0.001 versus Saline group. ###p ​< ​0.001 versus Peptide group. One-way ANOVA followed by the Bonferroni's test.

The FST and TST are two commonly used models to evaluate the preliminary antidepressant effects of drugs [23,24]. To investigate the potential antidepressant-like effect, the synthetic peptide (100 ​nmol) was i. t. administered to naïve mice 30 ​min prior to behavioral assays. As shown in Fig. 3, i. t. injection of the test peptide significantly reduced the depression-like behavior of mice in both the FST and TST, which could be blocked by naloxone.

### The tolerogenic potential of the test peptide

**Fig. 4:**
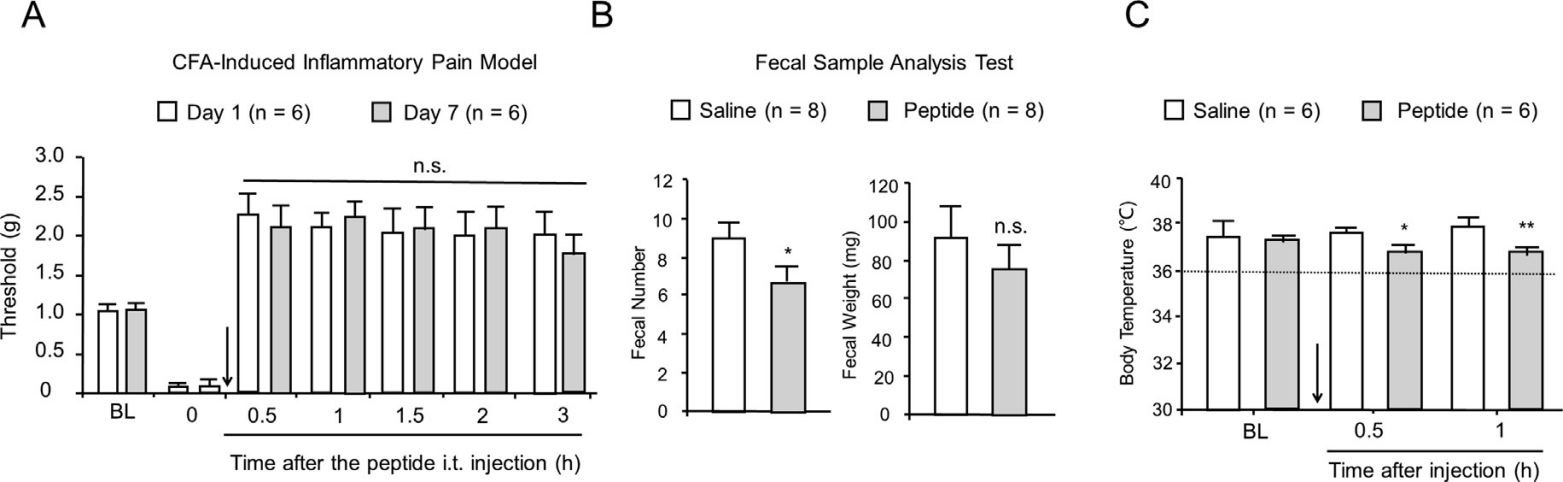
Evaluation of the adverse effects of the test peptide after i. t. injection. The dose of peptide is 100 nmol/mouse. (A) Antinociceptive tolerance. Mean ​± ​SEM, n ​= ​6. N.s., no significance. Day 7 versus Day 1, Two-way RM ANOVA followed by the Bonferroni's test. (B) Effect of the test peptide on gastrointestinal function. Mean ​± ​SEM, n ​= ​8. ∗p ​< ​0.05 versus Saline group, n. s., no significance. Student's t-test. (C) Body temperature study of the test peptide. Mean ​± ​SEM, n ​= ​6. ∗p ​< ​0.05 or ∗∗p ​< ​0.01 versus Saline group, Two-way RM ANOVA followed by the Bonferroni's test.

The tolerance potential of the test peptide was evaluated in a CFA-induced persistent inflammatory pain model. At the effective analgesic dose of the synthetic peptide, the CFA-treated model mice were dosed on seven successive days. Compared to Day 1, the peptide produced equivalent antiallodynia effect on Day 7 (Fig. 4A, F_1,10_ ​= ​0.0111, p ​= ​0.9182).

### The effect of the test peptide on gastrointestinal function

Furthermore, to evaluate the effect of the synthetic peptide on gastrointestinal function, a fecal sample analysis test was used in this study [20]. As shown in Fig. 4B, i.t. injection of the peptide (100 ​nmol) slightly inhibited gastrointestinal function (fecal number inhibition rate, 24%; fecal weight inhibition rate, 17%).

### Effect of the test peptide on thermoregulation

In the present study, the intrathecal administration of peptide (100 ​nmol) resulted in a modest reduction in body temperature among the intact mice (F_1,11_ ​= ​10.85, p ​= ​0.0072, Fig. 4C). However, the body temperature of the mice remained higher than 36 ​°C after the intervention, suggesting that this analgesic dose was insufficient to elicit hypothermia.

## Discussion

ORs belong to the G protein-coupled receptor family and are metabotropic receptors. At present, ORs participate in many processes, including pain modulation, mood regulation, endocrine function, and thermoregulation [25,26]. The *μ*OR is one of the most highly studied pharmacological targets for mitigating pain. However, the use of highly selective *μ*OR agonists can cause physical dependence, which can lead to abuse and addiction. An ideal opiate drug for pain relief should possess high analgesic efficacy without or with fewer adverse reactions, especially tolerance, respiratory depression threats, and drug dependence [27]. To circumvent opioid-related adverse effects, several strategies have emerged, including multitarget ligands [[28], [29], [30]], the generation of G protein-biased opioid agonists [31,32], and positive allosteric modulators of the *μ*OR [33,34].

Many studies have reported high comorbidity rates between depression and chronic pain, such as chemotherapy-induced neuropathic pain and cancer pain [35,36]. Comorbid depressive symptoms in chronic pain are troublesome and debilitating neuropsychiatric disorders, resulting in the worsening of chronic pain intensity, posing medication safety issues, reducing patient adherence, and wasting medical resources [37]. Effective pharmacological interventions to treat chronic pain and improve comorbidities are urgently needed.

The opioid system is also involved in modulating aspects of human mood, reward, and well-being [38]. Recently, the opioid system has regained interest in pain and comorbid disease studies due to the dysregulation of the opioid system in depression [39]. Pharmacological modulators of *μ*OR, *δ*OR, and *κ*OR may have antidepressant effects and the potential for the concomitant treatment of pain and depression [40]. This strategy has been effectively implemented through the use of the mixed opioid analgesic buprenorphine, an FDA-approved medication for the treatment of opioid addiction, chronic pain, and depression [14,41,42]. Previous studies also revealed that administration of the *μ*OR agonist morphine to rodents reduced depressive-like behaviors [43,44]. These antidepressant responses are caused by *μ*OR signaling-mediated euphoric effects [45].

The i.t. administration of analgesic medications offers a direct pathway to both the spinal cord and dorsal root ganglia, enabling drugs to circumvent the blood brain barrier and exhibit a more expedited and potent analgesic response than the systemic injection of medication (e.g., ziconotide). Consequently, we chose i.t. injection as the preferred route of administration for our study. In the present study, a novel peptide was found to produce antihyperalgesic and antidepressant-like effects in mice, resembling those of morphine. In naïve mice, the test peptide not only increased the basal pain threshold but also decreased the duration of immobility in both the FST and TST after i.t. administration. The potential antidepressant-like effect of the test peptide was blocked by naloxone, suggesting that the mechanism of action may be more closely related to its *μ*OR agonist effects [43]. The synthetic peptide produced profound and durable analgesia in a series of preclinical pain models, including spontaneous pain, inflammatory pain and persistent neuropathic pain. The spinal antihyperalgesic effect of the test peptide was completely reversed by naloxone, CTOP, NTI, or nor-BNI, suggesting that this effect of the peptide might be associated with direct or indirect effects on ORs.

A growing body of research has provided evidence for the involvement of OR signaling in the process of thermoregulation [[46], [47], [48]]. In a mouse model, *κ*OR activation generally results in heat loss [49], while *μ*OR activation results in opposite responses (heat gain) [47]. Currently, there is insufficient evidence to determine the involvement of *δ*ORs in thermoregulation. Numerous studies have reported that hypothermia, which is maladaptive and correlated with poor clinical outcomes, usually occurs within 1 ​h of dosing (e.g., morphine, i.t., 2 ​nmol; capsaicin, s. c., 9.8 ​μmol/kg) [21,50]; of note, thermoregulation in naïve mice was only marginally affected, and no hypothermia was found in the present study.

Chronic opioid exposure results in pharmacodynamic tolerance and is problematic for the effective control of pain. In our previous study, we confirmed that repeated i.t. injections of morphine at therapeutic doses significantly increase antinociceptive tolerance in a chronic inflammatory pain model [16]. In the present investigation, on the seventh day following repeated i.t. administration of test peptides, there was no statistically significant difference in the antihyperalgesic efficacy of the drug in the CFA model compared to that on the initial day of treatment.

Overall, given the favorable balance between beneficial effects (antihyperalgesic and antidepressant effects) and side effects resulting from the targeting of ORs at specific ratios, the use of multiple OR ligands may be a viable approach for developing multifunctional drugs to treat chronic pain and comorbid depressive symptoms.

In light of the ongoing opioid crisis, this study aimed to develop a specifically designed nonapeptide with potential therapeutic benefits for chronic pain and comorbid depressive symptoms by acting on multiple ORs. Although the detailed mechanism of action (e.g., receptor-binding capacity, selectivity, and agonistic/antagonistic properties) needs further in-depth research in the future, the obtained results support the feasibility of multitargies for the development of better-tolerated drug candidates. Multiple OR ligands potentially offer a promising alternative approach for mitigating chronic pain and simultaneously managing comorbidities associated with chronic pain (e.g., emotional disorders).

## Funding

This work was supported by the 10.13039/501100001809National Natural Science Foundation of China (82101302, 32070998, 32271054), the Foundation of 10.13039/501100002949Jiangsu Province “333 Project High-level Talents” (BRA2020076), 10.13039/501100002949Jiangsu Province innovation and entrepreneurship training program for college students (202310304039Z) and 10.13039/501100012246Priority Academic Program Development of Jiangsu Higher Education Institutions (10.13039/501100012246PAPD).

## Data availability

Data will be made available on reasonable request.

## Author contributions

**Yongjiang Wu**: Investigation, Data curation, Formal analysis, Writing–original draft. **Xiaofei Song**: Investigation, Data curation. **YanZhe Ji**: Investigation. **Gang Chen:** Validation, Writing–review & editing, Supervision, Project administration, Funding acquisition. **Long Zhao:** Conceptualization, Methodology, Validation, Formal analysis, Visualization, Writing –review & editing.

## Declaration of competing interest

The authors declare no conflict of interest.
